# *QuickStats:* Percentage* of Adults Aged ≥18 Years Living in Families That Were Food-Insecure in the Past 30 Days,^†^ by Family Income^§^ and Urbanicity^¶^ — National Health Interview Survey, United States, 2021**

**DOI:** 10.15585/mmwr.mm7148a6

**Published:** 2022-12-02

**Figure:**
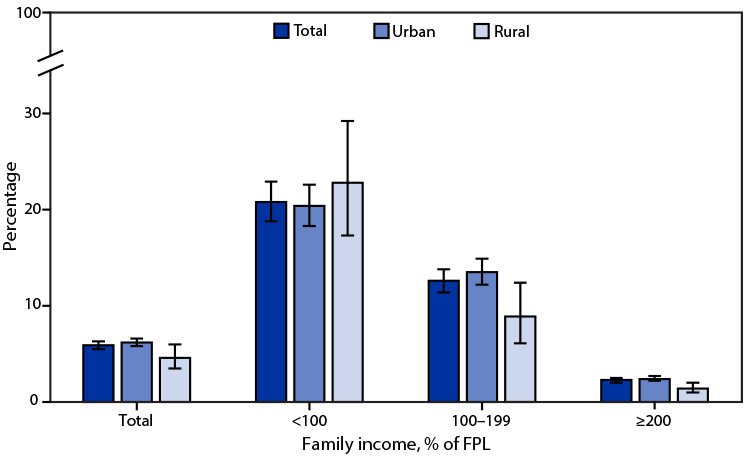
In 2021, 5.9% of adults aged ≥18 years lived in families that were food-insecure in the past 30 days. The percentage was higher in urban areas (6.2%) compared with rural areas (4.6%) overall and within households earning 100%–199% of FPL (13.5% versus 8.9%) and ≥200% of FPL (2.4% versus 1.4%). For adults living in families with incomes <100% of FPL, the percentage was similar in rural (22.8%) and urban (20.4%) areas. The percentage decreased with family income from 20.8% for those living in families earning <100% of FPL to 2.3% for those living in families earning ≥200% of FPL. The same pattern was found for adults living in urban and rural areas.

**Source:** National Center for Health Statistics, National Health Interview Survey, 2021. https://www.cdc.gov/nchs/nhis/index.htm

For more information on this topic, CDC recommends the following link: https://www.cdc.gov/chronicdisease/programs-impact/sdoh.htm

